# Isolated talonavicular arthrodesis and talonavicular-cuneiform arthrodesis for the Müller-Weiss disease

**DOI:** 10.1186/s13018-017-0581-4

**Published:** 2017-06-05

**Authors:** Hong-hui Cao, Wei-zhong Lu, Kang-lai Tang

**Affiliations:** 1 Department of Orthopaedic Surgery, Southwest Hospital, The Third Military Medical University, Gaotanyan Str. 30, Chongqing, 400038 People’s Republic of China; 2 Department of Orthopaedic Surgery, The Traditional Medical Hospital of Chongqing, China, The Brach 4th Panxi Road, Jiangbei, Chongqing, 400021 People’s Republic of China

**Keywords:** Müller-Weiss disease, Autoallergic iliac bone graft, Arthrodesis, Osteotomy

## Abstract

**Background:**

The study aimed to introduce the isolated talonavicular and talonavicular-cuneiform arthrodesis for the stage III and IV Müller-Weiss disease and analyze their clinical outcomes.

**Methods:**

Thirty patients of stage III and IV Müller-Weiss disease were divided into the talonavicular (TN) arthrodesis group and the talonavicular-cuneiform (TNC) arthrodesis group according to the perinavicular osteoarthritis by MRI scans. For the isolated talonavicular arthrodesis group, 16 patients underwent talonavicular arthrodesis with two 4.0 mm hollow headless compression screws. For the TNC arthrodesis group, 14 patients were received the TNC arthrodesis with reverse “V” shape osteotomy and autoallergic iliac bone graft. All patients were followed up at 3, 6, 9, and 12 months, and per 6 months after 1 year, by the AOFAS ankle-midfoot scores, and evaluated by radiographic measurements.

**Results:**

All of them were followed up in two groups and all patients were satisfied with their clinical results. At the TN arthrodesis group, the patients’ mean was 39.8 months (range, 11–66 months) follow-up. The mean AOFAS ankle and hindfoot scores had improved from 38.3 ± 5.1 preoperatively to 88.9 ± 1.9 at the last postoperative assessment. At the TNC arthrodesis group, the mean follow-up was 51.7 months (range, 12–90 months). The mean AOFAS ankle and hindfoot scores were 40.1 ± 7.9 preoperatively to 90.1 ± 2.0 at the last postoperative. All of the cases were solid fusion on the radiograph.

**Conclusions:**

According to MRI evaluation, either TN or TNC arthrodesis for stage III or IV Müller-Weiss disease have the good clinical outcomes with solid fusion rate and obvious improvement of the quality of life of patients.

## Background

Müller-Weiss disease is a primary osteonecrosis of the tarsal navicular of unknown etiology [1]. Several theories have been proposed, including primary osteonecrosis [2], traumatic or biomechanical causes, congenital malformation, navicular osteoarthritis [3], and abnormal evolution of Kohler’s disease [4], but the delayed ossification of the tarsal navicular and an abnormal force distribution pattern have been the most accepted [5]. Most of the patients complain of chronic dorsomedial midfoot pain on weightbearing midfoot pain resulting in perinavicular osteoarthritis.

Maceira [5] classified the patients into five stages according to lateral X-rays: stage 1 shows minimal changes, whereas stage 5 is defined by complete extrusion of the navicular, stages 2–4 showed different degree of the compression of the navicular and the lowering of the longitudinal arch height. But Maceira admitted that the severity of the symptoms may not correspond with the radiological destruction of navicular bone or the stage of the disease.

When prolonged conservative treatment fails, surgery may be indicated. We describe arthrodesis of talonavicular or talonavicular-cuneiform joint according to the perinavicular osteoarthritis by MRI scans for the stage III and IV Müller-Weiss disease. We believe the individualization arthrodesis can restore the length of the medial column and relieve the pain caused by removal between the fragments or osteoarthritis.

## Methods

**Fig. 1:**
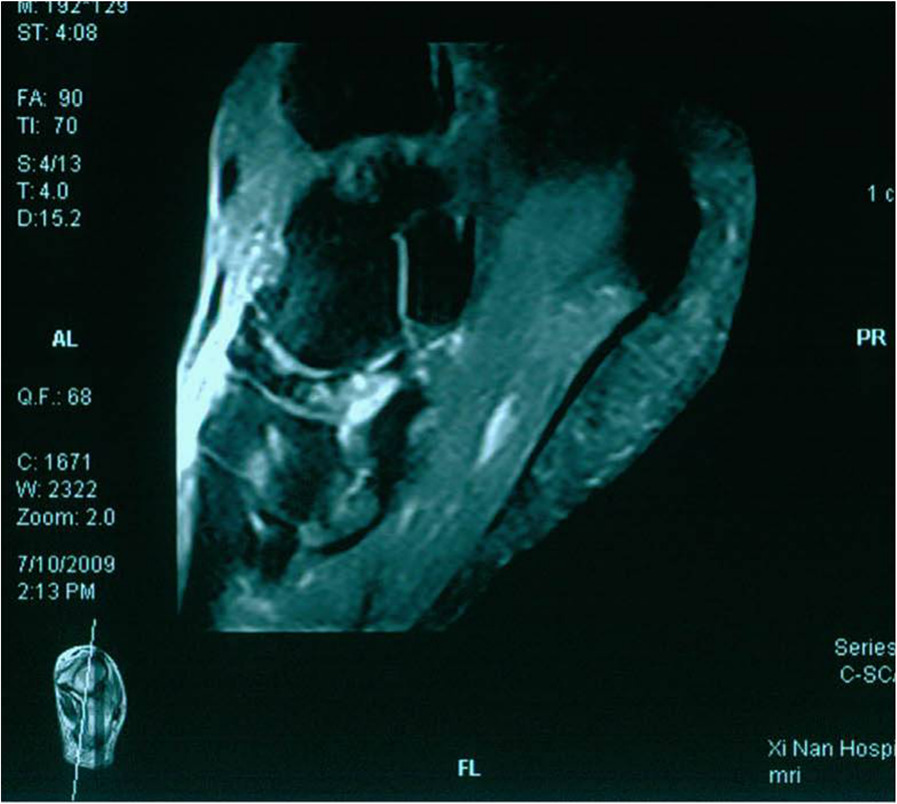
The necrosis of navicular and severe talonavicular joints arthritis in T2 fast-suppressed image of MRI scans

At the isolated talonavicular arthrodesis group, we records 16 patients since 2008 of the Southwest Hospital, Chongqing, China. Sixteen feet in 16 patients (2 men and 14 women) with MWD disease were identified. The average age of the patients at the time of surgery was 50.3 ± 8.4 years (range, 35 to 62 years). According to the Maceira classifications, there are 11 stage III and 5 stage IV. The compression of the navicular and the severe talonavicular joints arthritis were proved be processing in all cases by MRI (Fig. 1). But none of these patients showed obvious arthritic change in the calcaneocuboid or subtalar joint.

**Fig. 2:**
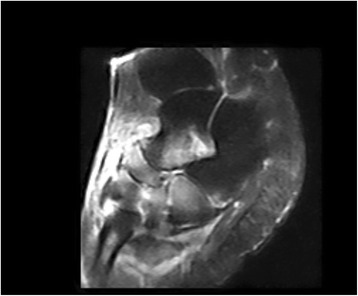
Magnetic resonance manifest osteonecrosis of the tarsal navicular bone and severe peri-navicular osteoarthritic changes in T2 fast-suppressed image

At the TNC group, we recorded 14 patients since 2008 at the same hospital. There involved 14 patients (4 men and 10 women) with the stage III MWD disease. The average age of the patients at the time of surgery was 49.2 years (range, 32 to 69 years). All patients complained of midfoot pain on standing and walking and had gradually collapsed of the medical longitudinal arch, with the arthritic change of talonavicular-cuneiform joint by MRI (Fig. 2).

In both groups, the patients were treated conservatively with insoles and physiotherapy for at least 6 months. None of them had trauma history, rheumatoid arthritis, renal failure, or lupus erythematosus. The patient, who accompanied with multiple arthritis or infection, obvious deformity in hindfoot, was excluded.

### The surgical technique

**Fig. 3:**
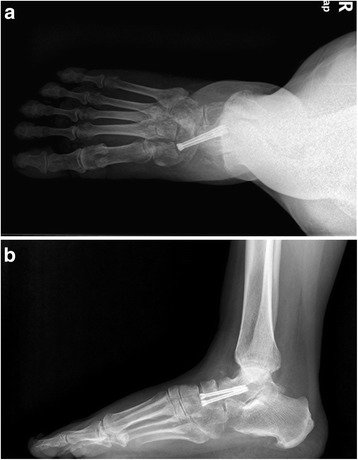
**a**, **b** Two 4.0 mm hollow headless compression screws were implanted through the talonavicular joint by vertically the articular surfaces

For the isolated talonavicular arthrodesis group, all patients underwent surgical intervention by the two senior authors (HH Cao and KL Tang) in our hospital. After general anesthesia, the procedure was performed in the supine position with a thigh tourniquet to stanch bleeding. An incision of about 4 cm was made between the anterior tibial tendon and the extensor hallucis longus to expose the talonavicular joint. The dorsolateral protruding necrotic navicular bone was excised and all residual cartilage was removed from the talonavicular joint. For the stage III cases, two 4.0 mm hollow headless compression screws (Newdeal, USA) were implanted through the talonavicular joint by vertically the articular surfaces (Fig. 3). The reverse “V” shape osteotomy of talonavicular joint to restore the medial arch height and autoallergic iliac bone graft were applied additionally for the stage IV cases.

**Fig. 4:**
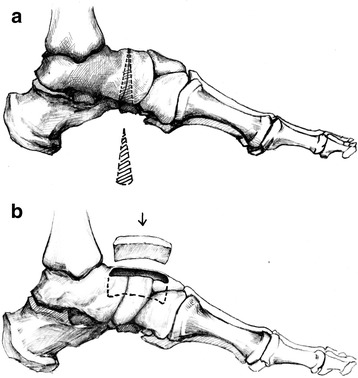
**a**, **b** The talonavicular joint was osteotomied by reversed “V” shape to restore the height of medial longitudinal arch (**a**). A tricortical autogenous graft was inserted in the rectangle bed which was carved on the dorsal side of talonavicular-cuneiform (**b**)

**Fig. 5:**
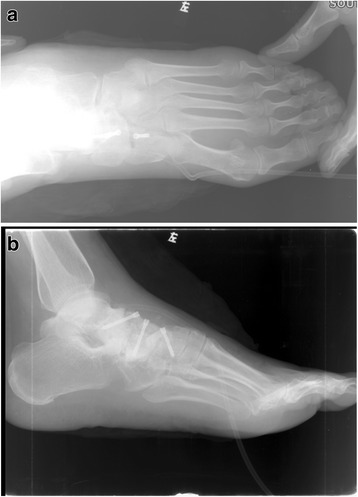
**a**, **b** Three cannulated titanium screws of 4.0 mm were implanted through the autogenous iliac bone into the triple bones across the talonavicular-cuneiform fusion interface

For the TNC arthrodesis group, the Prof. Tang performed all operations as the former description by us [6]. After removed the cartilage of the talonavicular-cuneiform articular surfaces, the talonavicular joint was osteotomied by reversed “V” shape to restore the height of medial longitudinal arch (Fig. 4a). And then, an about 3 cm × 1.5 cm × 0.5 cm rectangle bed is carved on the dorsal side of talonavicular-cuneiform. A tricortical autogenous graft of same size and shape as above described is obtained from the iliac crest and is inserted in the practiced bed with the aid of plantarflexion of the foot (Fig. 4b). Two or three cannulated titanium screws of 4.0 mm (Newdeal, USA) were implanted through the autogenous iliac bone into the triple bones across the talonavicular-cuneiform fusion interface (Fig. 5).

Fluoroscopic control was used throughout to ensure optimal placement of the hardware in the two groups.

### Postoperative management and evaluation

At the end of the surgery, a short leg cast was applied postoperatively for 6 weeks. The negative pressure wound drainage tube was removed within 48–72 h. Gradual protected weight bearing was allowed 6 weeks after surgery; walk with full weight bearing 3 months after surgery following radiographic evidence of consolidation.

The lateral and AP weight-bearing radiographs of foot were measured for evaluating the bone healing. All patients were evaluated pre-operatively, at 3, 6, 9, and 12 months, and per 6 months after 1 year by clinical examination with AOFAS ankle-hindfoot scores. The protocol of the foot pain, onset and deformity, and complications in or after surgery, were recorded exactly.

All the data was treated with SPSS 13.0, independent sample paired *t* test, and definite statistical difference as *p* < 0.05, significant statistical difference as *p* < 0.01.

## Results

All of them were followed up in two groups: 16 TN arthrodesis [age at the intervention, 50.3 years (range, 35–60years); follow-up, 39.8 months (range, 11–66 months); Maceira classifications, 12 stage III and 4 stage IV and 14 TNC arthrodesis [age at intervention, 49.2 years (range, 32–69 years); follow-up 51.7 months (range, 12–90 months); Maceira classifications, 14 stage III] (NS on all items between the two groups).

All patients were satisfied with their clinical results and were able to walk long distances 6 months after surgery in two groups. Only four patients in TNC arthrodesis complained the slight pain after long distance walking and can be ceased after rest or oral anti-inflammatory medication. The mean AOFAS ankle and hindfoot scores had improved from 38.1 ± 5.0 preoperatively to 88.1 ± 2.7 at the last postoperative assessment in the TN arthrodesis group (Table 1) and the TNC arthrodesis group is 40.1 ± 7.7 preoperatively to 90.2 ± 2.0 at the last postoperative (Table 2). All of two groups were significantly different between preoperative and postoperative. There is nothing significantly different between the two groups at the last postoperative assessment.

**Table 1:** Demographic description of the TN Arthrodesis patients

Patient No	Sex	Age	Stage	Side	Bone graft	Osteotomy	AOFAS Score	
							Preoperatively	Postoperatively
1	F	39	III	Left	no	no	35	80
2	M	39	III	Left	no	no	37	89
3	F	58	III	Left	no	no	33	88
4	F	54	III	Left	no	no	39	90
5	M	52	III	Left	no	no	32	90
6	F	41	IV	Left	yes	yes	39	87
7	F	60	IV	Left	yes	yes	34	89
8	F	57	III	Left	no	no	33	82
9	F	44	III	Left	no	no	47	87
10	F	62	IV	Left	yes	yes	35	90
11	F	50	III	Left	no	no	32	95
12	F	35	III	Left	no	no	55	89
13	F	69	IV	Left	yes	yes	41	92
14	F	37	III	Left	no	no	47	90
15	F	58	IV	Right	yes	yes	35	87
16	F	50	III	Right	no	no	35	84
Mean	—	50.3 ± 8.4	—	—	—	—	38.1 ± 5.0	88.1 ± 2.7

**Table 2:** Demographic description of the TNC Arthrodesis patients

Patient No	Sex	Age	Stage	Side	AOFAS Score	
					Preoperatively	Postoperatively
1	F	46	III	Left	55	92
2	F	52	III	Right	49	94
3	F	41	III	Right	26	88
4	M	58	III	Left	27	90
5	F	49	III	Left	32	86
6	F	37	III	Left	50	87
7	F	60	III	Left	34	89
8	M	47	III	Left	44	89
9	M	32	III	Right	44	90
10	F	62	III	Left	32	95
11	F	34	III	Left	50	89
12	F	50	III	Right	38	92
13	F	69	III	Left	40	90
14	M	52	III	Left	35	82
mean	—	49.2 ± 8.4	—	—	40.1 ± 7.7	90.2 ± 2.0

**Fig. 6:**
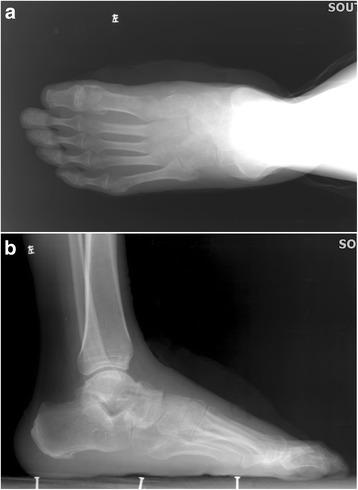
**a**, **b** One year later after post operation the TN interfixations have been removed and the TN joint fused solidly

**Fig. 7:**
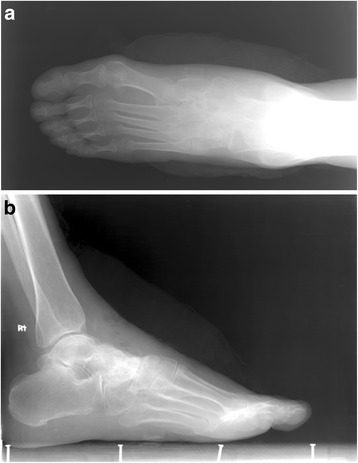
**a**, **b** The TNC joint have good solid fused after removing the TNC interfixation

Radiographically, All the operated feet fused solidly at 3 or 6 months after surgery without screw break or loosening; 9/16 in the TN arthrodesis group and 7/14 in the TNC arthrodesis group have removed the inter fixation (Figs. 6 and 7). All the operated feet fused solidly at 3 or 6 months after surgery without screw break or loosening. These results revealed improvements in terms of pain and mobility obtained by these surgical procedures.

## Discussion

Müller-Weiss disease is primary osteonecrosis of the tarsal navicular bone in adult and a rare pathology of unclear etiology in middle age population [1]. The diagnosis of MWD can usually be made with plain radiographs. Maceira [5] further describes the disease by developing a five-stage classification system using lateral weightbearing radiograph, showing a progressive collapse of the medial arch and compression and splitting of the tarsal navicular. It should be distinguished from Köhler’s disease [4], which occurs osteochondrosis of the tarsal navicular in children, and from secondary to systemic diseases (rheumatoid arthritis, SLE, renal failure, diabetes) or trauma [7].

It was reported that the therapy of MWD disease was described in the different stage [8]. For early MWD (stage I or stage II) with symptoms, initial conservative therapy includes ankle foot orthoses and oral anti-inflammatory medication. If conservative treatment failure, simple excision or drilling decompression is enough [9]. For moderate stage MWD (stage III or stage IV), isolated talonavicular arthrodesis and talonavicular-cuneiform (TNC) arthrodesis were reliable. MWD (stage V) with marked deformity, the surgical treatment should be double fusion or triple arthrodesis [8–10]. There is no gold standard surgical technique that is effective and safe for the treatment of MWD disease. But early diagnosis and proper treatment are essential for patients’ recovery [7]. Traditionally, the standard tools for the diagnosis of MWD disease and judgment of the stage are weightbearing plain radiographs of the foot. But it is difficult to show the minimal changes of adjacent joints. MRI scans may show loss of signal intensity of the navicular on theT1-weighted images and hyperintense diffuse marrow edema and periarticular fluid on the T2-weighted images [11]. In these two groups, we choose the isolated talonavicular arthrodesis and talonavicular-cuneiform (TNC) arthrodesis for the moderate MWD (stage III and IV) depending on the adjacent joints arthritic change by MRI scans. For the stage III or IV MWD disease, the isolated talonavicular arthrodesis is enough if the osteoarthrosis only exit in talonavicular joint. Talonavicular-cuneiform (TNC) arthrodesis is an advocated technique for the stage III MWD disease with alleviating the pain and excellent results in consolidation if osteoarthrosis in the joints around navicular. Reverse “V” shape osteotomy of the talonavicular joint can increase the medial longitudinal arch and avoid the secondary pain caused by flatfoot. We selected an allograft of tricortical iliac crest for reconstruction the excised navicular fragment [6].

The arthrodesis of peri-navicular could get the satisfaction clinic results. Lui [12] reported that of 6 patients with Müller-Weiss disease were treated with arthroscopic triple arthrodesis, at 43.5 months follow-up, the AOFAS ankle-hindfoot score increase 43.8 points (from 37.7 points preoperatively to 81.5 points postoperatively). CK Lu [11] reported that 13 feet of 13 patients with patients with Müller-Weiss disease with a mean age of 55.6 years were received the isolated talonavicular arthrodesis. The average modified American Orthopaedic Foot and Ankle Society (AOFAS) ankle-hindfoot score improved from 48.5 points preoperatively to 87.2 points at final follow-up (mean 51 months, range 10–114 months). Three cases were nonunion. In our study, either the isolated talonavicular arthrodesis or talonavicular-cuneiform (TNC) arthrodesis was gained the good clinic results and the solid fusion.

## Conclusions

Throughout, the main limitation of the current study is the small number of cases; either TN or TNC arthrodesis for stage III or IV Müller-Weiss disease according to MRI evaluation have the good clinical outcomes with solid fusion rate and obvious improvement of the quality of life of patients. A large sample size and long-term clinical follow-up studies are required to evaluate the efficacy and safety of these methods.
